# Comparison of the effect of mechanical massage and warm mechanical massage application on perceived labor pain and childbirth experience: A randomized clinical trial

**DOI:** 10.18332/ejm/132883

**Published:** 2021-02-27

**Authors:** Nükhet Kaçar, Neslihan Özcan Keser

**Affiliations:** 1 Ministry of Health, Ankara City Hospital, Maternity Hospital, Ankara, Turkey; 2 Department of Midwifery, Faculty of Health Science, Istanbul University-Cerrahpaşa, Istanbul, Turkey

**Keywords:** parturition, labor pain, midwifery, pregnancy, pleasure, massage

## Abstract

**INTRODUCTION:**

Birth is undisputedly one of the most painful experiences many women endure in their lives. This study aimed to compare the effects of mechanical and warm mechanical massage application in reducing labor pain and enhancing childbirth satisfaction in primipara women.

**METHODS:**

A randomized-controlled trial was conducted on 210 primipara women. Subjects were randomly divided into three groups (each group comprised 70 women) to receive either a mechanical massage, warm mechanical massage, or routine care (control). The intervention was applied twice on the lumbosacral section (relating to the back part of the pelvis between the hips) and pain level was assessed by using the visual analogue scale (VAS) before the intervention, immediately, half an hour and at 1 hour after intervention. The labor satisfaction level was assessed by using the childbirth experience questionnaire (CEQ) at 30 minutes postpartum.

**RESULTS:**

Comparing the intervention groups, there were no significant differences in terms of VAS scores in admission to hospital and immediately after the first intervention, but there were significant differences in terms of VAS scores at half an hour and an hour after the first intervention, and immediately, half an hour and 1 hour after the second intervention, and at 30th min postpartum. The pain level and mean duration of labor for each intervention group were found to be lower than the control group, and childbirth satisfaction score points were higher than the control group.

**CONCLUSIONS:**

Mechanical massage in the lumbosacral can be used as a reliable and effective method to reduce pain and increase childbirth satisfaction.

## INTRODUCTION

The International Association for the Study of Pain defines pain as an unpleasant sensory and emotional experience associated with actual or potential tissue damage or described in terms of such damage^1^.

Labor pain is a multidimensional subjective response to some sensorial stimulant that arises at the beginning of the labor process. In contrast to other acute and chronic pain experiments, labor pain is not related to pathology but to new life. Labor pain occurs in physiology, psychology and culture sociology of a woman^1-3^.

Past pain and labor experiences may have a significant effect on the next pregnancy. The positive effects of good management of labor pain may create an opportunity for next labor experiences to be positive^4^.

There are two different methods, pharmacologic and non-pharmacologic in labor pain management. In recent years, it is widely believed that birth is a natural physiological process and that it is preferrable to use non-pharmacological methods for pain management during labor. It is reported that besides showing less adverse effects, being simple and easy to implement, and requiring lower cost, than directly reducing pain, non-pharmacological methods such as assisting the woman in coping with labor pain and pain management are among the most important reasons why these methods are preferred^5-6^. Non-pharmacological methods reduce labor pain, women’s anxiety and fear, and contribute to the management of birth pain^2,7-18^.

According to the literature, yoga decreases stress and depression and increases awareness at childbirth^19^. Sterile water injection application and music decrease the pain^20-22^. Acupuncture and aromatherapy applications decrease labor pain and anxiety^23-25^. According to a systematic review, massage has an important role in reducing labor pain and delivery time, improving the sense of control and emotional experience of labour^26^. Studies show that good management of labor pain and implementation of non-pharmacologic methods reduce labor pain and delivery time and increase childbirth satisfaction^15,27^.

Labor pain is a major anxiety for pregnant women and their family. Labor pain management and reducing the cost of prepartum, intrapartum and postpartum care are significant in terms of midwifery care quality during _labor_^1,7,10,28^.

Massage applications can be applied in different ways such as cold, warm, on feet etc. This study used two different massage methods. These were mechanical and warm mechanical massage. The mechanical massage application was used to evaluate only mechanical influences of massage on labor pain. The warm mechanical massage application was used to evaluate mechanical and warmth influences on labor pain.

This study aimed to compare the effects of mechanical massage and warm mechanical massage applications in reducing labor pain and enhancing childbirth satisfaction in primipara pregnant women.

## METHODS

### Study design and setting

This randomized controlled trial included 210 pregnant women referred to a hospital in Turkey (November 2018 to April 2019).

### Participants

This randomized controlled trial (n=120) included three groups (mechanical massage, warm mechanical massage, and control group). The pregnant women were invited to take part in the study. Those who accepted to participate, filled in the informed consent form. Due to ethical considerations, permission was obtained from the hospital located in Ankara and the ethics committee in Istanbul. The Permission Slip Number is 35640939-799, obtained from the Ministry of Health, Ankara, Turkey. The Ethical Approved Number is 59491012-604.01.02 obtained from the Clinical Research Ethics Committee, İstanbul University-Cerrahpaşa, İstanbul, Turkey.

**Figure 1:**
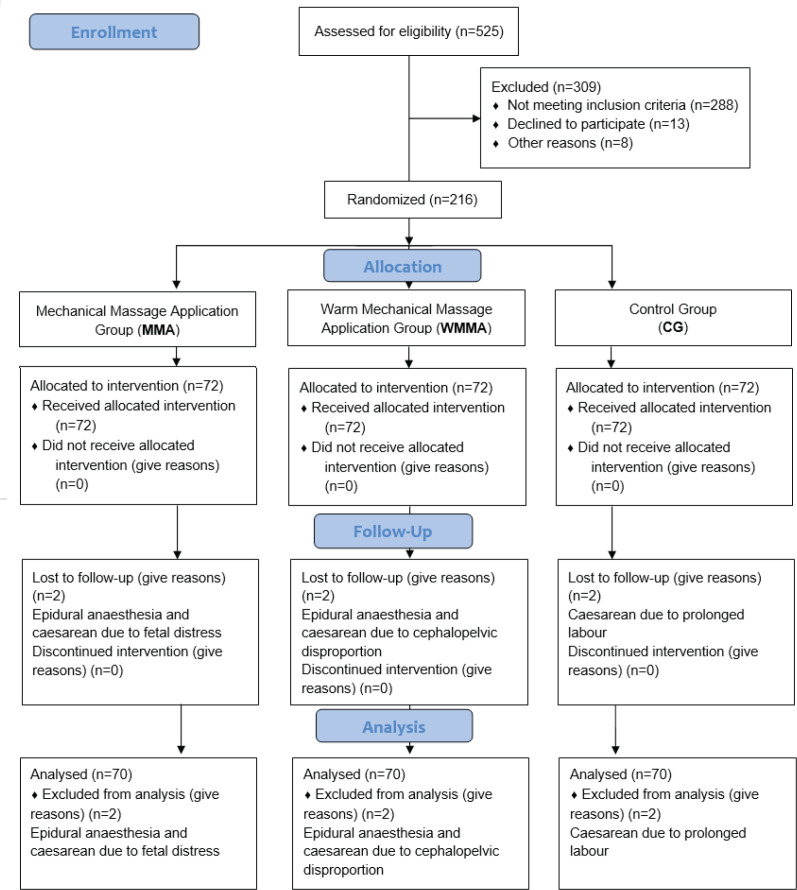
CONSORT flow diagram of participants

The CONSORT 2010 flow diagram was used, which provided randomization among participants^29^. According to The CONSORT 2010 flow diagram^29^, there were 525 volunteers, and each was interviewed. Of these, 309 of participants were excluded from the study because they did not meet the inclusion criteria (288), declined to participate (13) or other reasons (8). The rest, 216 participants, were randomized and three groups were created, each with 72 women. Eventually, 6 participants dropped out; because of epidural anesthesia (2); and caesarean section (4) due to cephalopelvic disproportion, fetal distress and prolonged labor (Figure 1).

Inclusion criteria were: women who were primipara, aged 20–30 years, had a singleton and term pregnancy (37–42 weeks), pregnancy process with no complications, vertex presentation of fetus, estimated fetal weight 2500–4000 g, cervical dilatation ≤4 cm, in order to evaluate the labor pain level at admission to hospital and before massage application, and to specify the starting point for all pregnant women who participated in the study.

Subjects that had gestational diabetes, pre-eclampsia/eclampsia, hyperemesis gravidarum, hemorrhage, malpresentation, multiple pregnancy, communication impairments, objection to massage, smoked, or received antenatal training from any hospital, were excluded. In addition, any complications during the labor such as pharmacologic drugs use, caesarean section or death of the baby led to exclusion.

### Instruments

#### Demographic questionnaire

The questionnaire consisted of 11 questions on age, marriage duration, education level, working status, welfare level, social security, number of pregnancies, miscarriage, curetting, dysmenorrhea, and doctor checks.

#### Visual analog scale (VAS)

The visual analog scale (VAS) was used to assess pain severity. It is a reliable tool that has been used in various studies to determine pain intensity on a scale 0–10. This scale has been used in different studies, and its reliability and validity have been confirmed^30-32^. High scores indicate more severe pain.

#### Childbirth experience questionnaire (CEQ)

A Likert scale was used to assess the satisfaction of childbirth. The scale consisted of 22 items. The first 19 items were evaluated using a 4-point Likert scale and the last 3 items were evaluated using VAS. Higher scores indicate an increased level of satisfaction of childbirth. Mamuk and Davas^11^ translated the scale into Turkish and performed validity and reliability studies. Cronbach’s alpha reliability coefficient of subscales was 56–76. This scale was reliable in the current study; the internal reliability consistency of subscales was found to be 55–82.

#### Questionnaire form of mechanical and warm mechanical massage application

This questionnaire form, consisting of 4 questions, was developed by the present researchers. Its aim is to evaluate the response of pregnant women to massage application. These questions investigated massage application satisfaction, effective labor pain reduction, willingness to suggest the massage application to other pregnant women, and request for massage application in the delivery room. There are five responses possible to each of the 4 questions, graded 1–5 points. A negative answer was given 1 point and a positive response scored a maximum of 5 points, according to the positiveness degree. Increasing points show that the participants chose positive answers to massage satisfaction, effectiveness of massage, willingness to suggest the massage, and requesting the massage.

### Procedure

We asked the pregnant women whether they wanted to participate in the study and obtained consent forms from those who accepted to participate in the study. The participants were randomly allocated into three groups: mechanical massage application, warm mechanical massage application, and control.

#### The procedure of mechanical massage application group

In previous studies, it was reported that massage application commenced at active phases of labor and was performed on average every 20 minutes, and subjects indicated that the contraction interval times had gradually decreased^5,33-38^. Hence in the present study, massages were applied to the intervention groups after the cervical dilatation became 4–5 cm and 7–8 cm and continued approximately for 15 min.

The demographic questionnaire was filled in by the participating pregnant women. We evaluated labor pain level via VAS at admission to hospital, examined the cervix, and registered the findings.

When cervical dilatation was 4–5 cm, the first mechanical massage application was performed for 15 min using a massage glove on the lumbosacral region. The pregnant women direct where to do the massage. At the end of the first post intervention, we evaluated the labor pain level via VAS immediately, half an hour later, and an hour later, and registered pain levels. When cervical dilatation was 7–8 cm, we applied a second mechanical massage application using a massage glove, as with the first intervention. Subsequently, at the end of the second post intervention, we evaluated the labor pain level via VAS immediately, half an hour later and an hour later, and registered pain levels. We applied the massage application only two times and did not apply massage again, and we accompanied the pregnant woman till birth occurred. After the pregnant woman gave birth, we evaluated the labor pain level via VAS and evaluated childbirth satisfaction using the CEQ based on the study of Mamuk and Davas^11^ at 30 minutes postpartum and the findings were registered. At the 30th minute after birth, we evaluated the pain, which results from both uterus involution and occurring uterus involution during the mother breastfeeding her baby.

Afterwards, midwifery care was given such as skin-to-skin contact, breastfeeding etc., and we evaluated massage satisfaction using the questionnaire form of mechanical massage application.

The massage glove is an instrument suitable for multiple uses, and easily available on the internet. It can be worn like a glove and has metal balls inside. The massage glove consists of 9 metal balls, each of 1.5 cm diameter and can rotate 360 degrees.

#### The procedure of warm mechanical massage application group

The demographic questionnaire was filled in by participating pregnant women. We evaluated the labor pain level via VAS at admission to hospital, examined the cervix, and registered the findings. We applied a procedure just as we did for mechanical massage application. At the intervention, a cherry-pit pack instead of a massage glove was used as a massage device. Before using the cherry-pit pack, it was heated for 2–3 min in the microwave oven.

#### The procedure of control group

The demographic questionnaire was filled in by the participating pregnant women. We evaluated the labor pain level via VAS at admission to hospital, examined the cervix, and registered the findings.

One of the researchers working at the hospital and performing the study gave the control group standard midwifery care. Within standard midwifery care, the researcher accompanied the mother intermittently, examined the cervix, measured the fetal heart rate, examined the mother’s vital signs and whether the amniotic sac was ruptured. Additionally, the researcher gave mothers regime 1 or 2, including water, compote, juice etc. No pharmacological drugs were used. Non-pharmacological methods such as massage, acupuncture, hypnotherapy, music etc., were not applied.

We evaluated the labor pain level via VAS immediately, half an hour later and an hour later, after cervical dilatation became 4–5 cm and registered the pain levels. We did not re-evaluate the labor pain level till cervical dilatation became 7–8 cm. Subsequently, we assessed the labor pain level via VAS immediately, half an hour later and an hour later after cervical dilatation became 7–8 cm and registered the pain level. A researcher accompanied the pregnant woman till birth occurred. After the birth, we evaluated the labor pain level via VAS and evaluated labor satisfaction using the CEQ based on the Mamuk and Davas^11^ study at 30 minutes postpartum and the findings were registered. Afterwards, midwifery care was given such as skin-to-skin contact, breastfeeding etc., and we evaluated massage satisfaction using the questionnaire form of mechanical massage application.

### Statistical analysis

The data were analyzed in SPSS for comparisons between the groups. While one-way ANOVA and chi-squared tests were used for comparison of sociodemographic and obstetric variables, Student’s t-test was used for comparison of mechanical massage and warm mechanical massage applications.

## RESULTS

There were no significant differences between the three groups in terms of sociodemographic characteristics and obstetric characteristics (Table 1).

**Table 1:** Sociodemographic and obstetric characteristics of three groups (N=70)

*Characteristics*	*Mechanical massage (A) Mean±SD*	*Warm mechanical massage (B) Mean±SD*	*Control (C) Mean±SD*	*F*	p*
Age (years)	23.49±2.95	24.50±3.05	24.26±3.26	2.04	0.31
Marriage duration (months)	19.97±12.37	19.09±9.54	19.40±12.84	0.10	0.90
	***%***	***%***	***%***	***χ^2^***	p*
Participants with high school diploma or higher	62.9	45.8	72.8	13.50	0.19
Participants do not have any job	87.1	94.3	92.9	2.55	0.27
Participants have good or better welfare	90.0	88.6	87.1	2.18	0.70
Participants have social security	82.9	84.3	84.3	0.07	0.96
Primigravida	84.3	81.4	87.1	0.86	0.65
Participants do not have abortion	90.0	90.0	94.3	1.09	0.57
Participants do not have curettage	92.9	91.4	92.9	0.13	0.93
Participants seeing a doctor during pregnancy	87.1	91.4	85.7	4.90	0.08

* The p values are for all three groups: A, B and C. Data were analyzed with one-way ANOVA. SD: standard deviation.

For the intervention group, there were no significant differences in terms of VAS scores in admission to hospital and immediately after first intervention, but there were significant differences in terms of VAS scores at half an hour and an hour after first intervention, and immediately, half an hour and an hour after the second intervention, and 30th min postpartum. Mechanical massage application was more effective in reducing labor pain and increasing childbirth satisfaction than warm mechanical massage application. Comparisons of the intervention groups and control group showed that there were no significant differences with regard to admission to hospital but other evaluations had significant differences (Table 2).

**Table 2:** Comparison of VAS scores of three groups (N=70)

*Stages*	*VAS scores Mean±SD*	*Mechanical massage application (A) p*	*Warm mechanical massage application (B) p*	*Control group (C) p*	*Groups*
**Admission to hospital**					
A	1.07±0.74	-	0.779	0.698	
B	1.03±1.03	0.779	-	0.558	A,B,C
C	1.13±0.89	0.698	0.558	-	
**Immediately 1st post-intervention**					
A	2.14±0.82	-	0.215	<0.001	
B	2.31±0.80	0.215	-	<0.001	A,B<C
C	3.21±1.19	<0.001	<0.001	-	
**Half an hour 1st post-intervention**					
A	2.73±1.03	-	0.027	<0.001	
B	3.13±1.07	0.027	-	<0.001	A<B<C
C	4.11±1.13	<0.001	<0.001	-	
**An hour 1st post-intervention**					
A	3.40±1.17	-	0.009	<0.001	
B	3.91±1.11	0.009	-	<0.001	A<B<C
C	4.80±1.22	<0.001	<0.001	-	
**Immediately 2nd post-intervention**					
A	4.30±1.20	-	<0.001	<0.001	
B	5.10±0.93	<0.001	-	<0.001	A<B<C
C	6.83±0.96	<0.001	<0.001	-	
**Half an hour 2nd post-intervention**					
A	4.54±1.28	-	<0.001	<0.001	
B	5.91±1.26	<0.001	-	<0.001	A<B<C
C	7.66±0.93	<0.001	<0.001	-	
**An hour 2nd post-intervention**					
A	5.49±1.39	-	<0.001	<0.001	
B	6.77±1.28	<0.001	-	<0.001	A<B<C
C	8.57±1.01	<0.001	<0.001	-	
**30th min postpartum**					
A	1.10±0.61	-	<0.001	<0.001	
B	1.49±0.58	<0.001	-	<0.001	A<B<C
C	2.39±1.04	<0.001	<0.001	-	

VAS: visual analog scale. Data were analyzed with independent sample t-test. SD: standard deviation.

There were statistically significant differences between the intervention groups and the control group in terms of childbirth satisfaction. Intervention groups received more points for CEQ than the control group (Table 3).

**Table 3:** Mean point in labor satisfaction of three groups (N=70)

*Subdimension groups*	*VAS scores Mean±SD*	*Mechanical massage application (A) p*	*Warm mechanical massage application (B) p*	*Control group (C) p*	*Groups*
**Labor process**					
A	2.97±0.32	-	0.090	<0.001	
B	2.88±0.24	0.090	-	<0.001	A,B>C
C	2.46±0.33	<0.001	<0.001	-	
**Professional help/support**					
A	3.54±0.52	-	0.813	<0.001	
B	3.56±0.46	0.813	-	<0.001	A,B>C
C	2.11±0.68	<0.001	<0.001	-	
**Perceived security/memories**					
A	3.16±0.32	-	0.126	<0.001	
B	3.09±0.21	0.126	-	<0.001	A,B>C
C	2.45±0.37	<0.001	<0.001	-	
**Agree with the decisions**					
A	2.49±0.58	-	<0.001	<0.001	
B	3.34±0.55	<0.001	-	<0.001	B>A>C
C	1.33±0.48	<0.001	<0.001	-	-

VAS: visual analog scale. Data were analyzed with independent sample t-test. SD: standard deviation.

There were no statistically significant differences between the intervention groups in terms of massage application satisfaction. However, there were statistically significant differences in terms of reducing labor pain, recommending the massage application to other pregnant women, and requesting the use of massage application in the delivery room (Table 4). Also, 64.3% of all participants requested the massage application from a midwife.

**Table 4:** Mean received points for questions of massage application of intervention groups (N=70)

*Group question*	*Mechanical massage (A) Mean±SD*	*Warm mechanical massage (B) Mean±SD*	*t*	*p*
Massage application satisfaction	4.86±0.35	4.93±0.49	-0.989	0.325 A,B
Effect on reducing labor pain	4.73±0.44	4.97±0.16	-4.24	<0.001 A< B
Recommending massage application for other pregnant women	4.70±0.49	4.90±0.30	-2.89	0.004 A<B
Requesting the use of massage application in delivery room	4.86±0.42	5.00±0.00	-2.80	0.006 A<B

Data were analyzed with independent sample t-test. SD: standard deviation.

Comparisons between active to transition phase and transition phase to childbirth, there were no significant differences between the intervention groups and control group in terms of the mean time between 4–5 cm and 7–8 cm cervical dilatation. However, there were statistically significant differences between the intervention groups and control group in terms of the mean time between 7–8 cm cervical dilatation and childbirth. The intervention groups had a shorter transition phase than the control group (Table 5).

**Table 5:** Mean time (minutes) in active phase and transition phase of intervention groups (N=70)

*Phases groups*	*VAS scores Mean±SD*	*Mechanical massage application (A) p*	*Warm mechanical massage application (B) p*	*Control group (C) p*	
**Between 4–5 and 7–8 cm cervical dilatation** (min)					
A	214.70±116.25	-	0.24	0.532	
B	180.54±46.07	0.24	-	0.137	A,B,C
C	202.53±113.71	0.532	0.137	-	
**Between 7–8 cm cervical dilatation and childbirth** (min)					
A	118.24±61.05	-	0.888	0.003	
B	117.06±34.97	0.888	-	<0.001	A,B<C
C	149.13±59.29	0.003	<0.001	-	

VAS: visual analog scale. Data were analyzed with independent sample t-test. SD: standard deviation.

## DISCUSSION

The results showed that mechanical and warm mechanical massage application on the lumbosacral area significantly reduced labor pain intensity immediately, half an hour and an hour following the interventions. These findings were in accordance with previous studies which recommended massage as an effective, non-invasive and easy to use technique in labor pain relief^10,35,39-41^.

A systematic review and meta-analysis by Ranjbaran et al.^42^ recommended massage for effective labor pain relief. Mortazavi et al.^36^ applied massage at 3–4, 5–7 and 8–10 cm cervical dilatation and found it to be effective in the relief of labor pain and anxiety and produced higher massage satisfaction^36^. Yildirim et al.^43^. applied the pressure on the LI4 point and found that the massage effective in labor pain relief. Sanli et al.^44^ applied foot massage at 4–5, 6–7 and 8–9 cm for 20 min, and an assessment of the massage application 10 min afterwards and found that it had reduced labor pain. In other studies, it was found that ice massage was effective in labor pain relief^33,45,46^. In another study, it was found that massage reduced labor pain immediately, at half an hour and an hour after the intervention^34^. Comparisons between massage application and other methods such as acupressure, music therapy, position change and hot compress, show that massage application is more effective in reducing labor pain^5,47-49^. In the systematic review by Smith et al.^26^, although it was reported that the applied massage group had lower labor pain intensity, there were no significant differences with other methods in terms of control in labor, augmentation ratio, and delivery time.

The main objective of our study was to compare mean labor pain intensity and childbirth satisfaction, between two different interventional methods and a control group. The results indicated that there were differences between the three groups in terms of VAS scores and childbirth satisfaction.

Mechanical massage application was more effective in reducing labor pain than warm mechanical massage application. It is believed that this difference is due to the use of a glove in mechanical massage application, as the massage glove has a straighter outline, it is practicable and firmer than a massage pillow, which is more difficult to apply.

The intervention groups were more satisfied with the childbirth than the control group. We believe that the reason for this difference is that, while a midwife gave the mothers in the intervention group continuous care due to massage applications, she intermittently gave the mothers in the control group standard midwifery care.

Warm mechanical massage application had a higher level of satisfaction than the mechanical massage application because of the use of a warm pillow (warmed cherry-pit pack).

## CONCLUSIONS

Mechanical massage application was more effective in reducing labor pain and increasing childbirth satisfaction than the application of a warm mechanical massage. At the same time the intervention groups scored higher in less labor pain and childbirth satisfaction than the control group. Our findings suggest that mechanical massage application reduces labor pain and increases childbirth satisfaction and should be considered as a non-invasive, inexpensive, simple and easily available method. The massage applications have the advantage of no adverse effects, and midwives can perform them confidently.
